# Molecular Analysis of PIK3CA in Metastatic Hormone Receptor-Positive Breast Cancer in Chile: Clinical and Pathological Insights

**DOI:** 10.3390/ijms252212246

**Published:** 2024-11-14

**Authors:** Carla Araya, Bárbara Mino, Patricio Le Cerf, Fancy Gaete, Ricardo Armisen, Daniel E. Carvajal-Hausdorf

**Affiliations:** 1 Clínica Alemana Facultad de Medicina, Universidad del Desarrollo, Santiago 7650567, Chile; carlaraya@udd.cl (C.A.);; 2 Doctorado en Ciencias e Innovación en Medicina, Universidad del Desarrollo, Santiago 7610658, Chile; 3 Servicio de Anatomía Patológica, Hospital Dr. Sótero del Río, Santiago 7610658, Chile; 4 Servicio de Anatomía Patológica, Hospital Dr. Luis Tisné B, Santiago 7930426, Chile; 5 Instituto de Ciencias e Innovación en Medicina, Universidad del Desarrollo, Santiago 7610658, Chile

**Keywords:** metastatic hormone receptor-positive breast cancer, PIK3CA mutations, HER2-low

## Abstract

Breast cancer is the most common cancer among women and a leading cause of cancer-related deaths. PIK3CA gene mutations, which are often present in advanced HR+ breast cancer, can be targeted by alpelisib. However, data on PIK3CA mutations in Chile are limited. Here, we aim to assess the mutational status of PIK3CA in metastatic breast cancer tissues from Chilean patients and describe their clinicopathological characteristics and survival outcomes. We analyzed 102 formalin-fixed, paraffin-embedded metastatic breast cancer samples from 96 patients diagnosed at three Chilean hospitals between 2007 and 2023. PIK3CA mutations were identified using targeted sequencing, and clinicopathological data were collected. We evaluated associations between mutational status, clinicopathological features, and survival. The median age at diagnosis was 56 years. The most common metastatic sites were liver (29.4%), bone (17.6%), and lung/pleura (16.7%). Most patients were HR+ HER2− (83.3%), with 57.3% showing HER2-low status. PIK3CA mutations were present in 40.6% of patients, mainly in exons 7, 9, and 20. No significant associations were found between PIK3CA mutations and clinicopathological characteristics or survival. Our study reveals a high frequency of PIK3CA mutations in HR+ metastatic breast cancer, consistent with global data. The majority of mutations are targetable with alpelisib. The proportion of HER2-low status patients suggests potential benefits from novel HER2-targeted therapies. These findings highlight the need for routine molecular diagnostics in Chile to improve personalized treatment and address economic and access challenges.

## 1. Introduction

Breast cancer is the most common cancer worldwide and the leading cause of cancer-related mortality in women [1]. In Chile, the incidence and mortality rates are 57.8 and 18.2 per 100,000 women per year, respectively (WHO cancer statistics, http://globocan.iarc.fr/; accessed September 2024). The peak incidence of invasive breast cancer diagnosis occurs in women aged 65 to 80, but it is also detected in younger women under 50, with an increasing incidence in recent years [1]. About 80% of diagnosed cases are invasive carcinomas, with 60–80% being hormone receptor-positive breast cancers (estrogen and progesterone) [2]. Despite a generally better prognosis, over 30% of patients will progress to metastatic disease [3]. In these cases, an organ-specific dissemination pattern is observed, preferentially affecting the bone (47–60%), liver (19–20%), lung (16–34%), and brain (10–16%), with a median survival of up to 46.7 months [4]. The 5-year survival rate can be as low as 20%. A study by Mariotto in 2017 estimated that more than 150,000 women in the United States were living with stage IV or metastatic breast cancer [5], with three out of four of them initially diagnosed at an early stage. With currently available treatment lines, a median overall survival of up to 52.2 months has been reported [6] in the context of clinical trials. In Chile, there is no epidemiological data on post-diagnosis follow-up or treatment of metastatic patients. The only study published in 2020 reports a median overall survival of 37 months (n = 221) in hormone receptor-positive breast cancer patients [7].

PIK3CA is the most frequently mutated gene in hormone receptor-positive breast cancer [8]. It encodes the catalytic subunit of the PI3K protein [9]. When a mutation activates this protein, there is an increase in signaling through the AKT-mTOR pathway, promoting tumor cell survival, proliferation, growth, and motility [10]. In May 2019, the United States Food and Drug Administration approved the use of alpelisib [11], a PI3K inhibitor, in combination with fulvestrant for postmenopausal patients with hormone receptor-positive metastatic breast cancer and mutations in exons 7, 9, and 20 of the PIK3CA gene. This regimen is indicated after progression during or after receiving aromatase inhibitors (in combination with CDK 4/6 inhibitors), according to the SOLAR-1 study [12]. Detecting these mutations requires molecular testing, which is unavailable in the Chilean public healthcare network. In this study, we aimed to determine the mutational status of the PIK3CA gene in metastatic tissues from a series of patients with advanced hormone receptor-positive breast cancer diagnosed at three tertiary hospitals in Chile. Secondarily, we described their clinicopathological characteristics and survival.

## 2. Results

The clinicopathological characteristics of the patients are summarized in Table 1. The median age of the patients was 56 years at stage IV (range: 36–85 years). At diagnosis, 67.7% (65/96) of patients were 50 years or older, and 24% (23/96) were metastatic. The median follow-up after the diagnosis of metastasis was 28.7 months (range: 0.3–181.3 months). The most common metastatic sites were liver (29.4%, N = 30/102 samples), bone (17.6%, N = 18/102 samples), and lung/pleura (16.7%, N = 17/102 samples). The predominant immunohistochemical pattern observed was hormone receptor-positive, HER2-negative (HR+ HER2−; 83.3% of patients (80/96)). HR+ HER2+, HR− HER+, and HR− HER2− patterns had frequencies of 6.3% (6/96 patients), 4.2% (4/96 patients), and 6.3% (6/96 patients), respectively. Among HER2− patients, 57.3% (55/96 patients) were HER2-low (immunohistochemistry score 1+ and 2+, not amplified), and 18.8% (18/96 patients) were HER2-ultralow (immunohistochemistry score 0).

**Figure 1:**
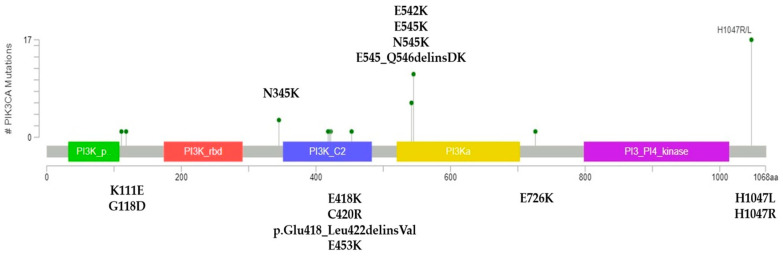
Overview of PIK3CA mutations in the Chilean MHRPBC series. Lollipop plot showing the distribution and quantity of PIK3CA mutations in this series. Created using MutationMapper (accessed from cBioPortal).

**Figure 2:**
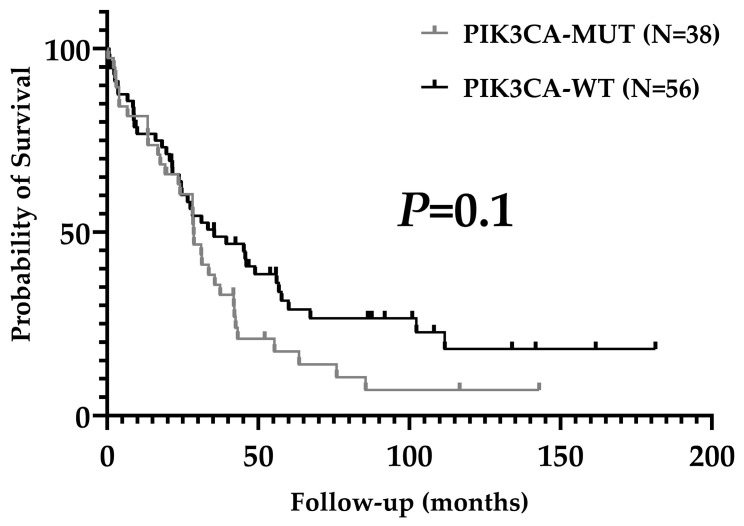
Overall survival according to PIK3CA mutational status.

Average coverage for sequenced regions of PIK3CA was 926X (range 340–2000). In our series, 40.6% of patients presented pathogenic PIK3CA mutations (39/96). Substitutions were present in 37/39 patients (94.9%) and indels in 2/29 (5.1%). Mutations in exons 7, 9, and 20 were observed in 35.4% of patients (34/96) and corresponded to 87.2% of all mutations. Five patients had mutations in exons 1 and 4 (5.2%). Three patients had two concurrent pathogenic mutations (3.1%). The distribution and quantity of mutations in the PIK3CA gene are shown in Figure 1. H1047R (exon 20), E545K (exon 9), E542K (exon 9), and H1047L (exon 20) were the most common mutations detected (28.2%, 20.5%, 15.4%, and 15.4% of patients, respectively). PIK3CA mutational status was not associated with clinicopathological characteristics (Table 2) or overall survival (Figure 2).

Finally, we extracted data from the MBC Project repository in cBioPortal, including 70 hormone receptor-positive breast cancer patients with known progression to a metastatic stage (Table 3). Most common metastatic sites (ever) were bone (68.6%), liver (35.7%), and lung/pleura (31.4%). Nineteen patients had oncogenic PIK3CA mutations (27.1%). No patients had concurrent pathogenic variants in PIK3CA. The most frequent mutations were detected in exon 9 (57.9%) and exon 20 (36.8%). No mutations were found in exon 7.

## 3. Discussion

In this study, we used a retrospective series of advanced hormone receptor-positive breast cancer and determined their PIK3CA mutational status, clinicopathological characteristics, and survival. Over 40% (34/96) of patients harbored pathogenic mutations in this gene. Moreover, almost 90% of them could be candidates for targeted treatment with alpelisib.

Our results are concordant with previous reports. Earlier studies showed variable occurrence (25–46%) of PIK3CA mutations in metastatic ER+ breast cancer [13,14,15,16,17,18,19,20]. More recently, Rugo et al. [21] found 35% of advanced HR+ HER2-negative patients had PIK3CA mutations, with 80% having exon 7, 9, and 20 alterations with on-label indication for alpelisib. Additionally, they reported similar mutation frequencies in matched tissue and liquid biopsies, a finding also shared by Suppan et al. [22]. Similarly, Reinert et al. [23] showed that 37.5% of Brazilian ER+ HER2− metastatic patients harbored PIK3CA mutations, from which 78% were included in a companion diagnostic test for alpelisib. Using a similar methodology, almost 50% of advanced HR+ HER2− patients with resistance to palbociclib plus aromatase inhibitors or fulvestrant showed pathogenic PIK3CA mutations in a Korean cohort [24] Kindt et al. [25] recently showed 80% PIK3CA-mutated, circulating DNA in patients with resistance to CDK4 inhibitors plus endocrine therapy, though with a small sample size. In Chile, a study by Garrido et al [26] in advanced solid tumors showed 50% of PIK3CA mutations in 16 metastatic breast cancer patients. However, HR status was not shown.

We searched for a publicly available database containing comparable patients to our series. However, data are still lacking. We accessed information from the Metastatic Breast Cancer Project [27] and found 70 patients with hormone receptor-positivity and known progression to metastatic disease. We found a similar profile of metastatic sites and a predominance of exon 9 and 20 mutations. However, the frequency of oncogenic PIK3CA mutations was lower (27.1% vs. 40.6%), even for estrogen receptor-positive primary tumors [8,28].

Another interesting result among our HR+ HER2 patients was that 57.3% had HER2-low status. These patients are known to benefit the most from therapy with trastuzumab deruxtecan [29], and detection methods used in clinical trials are already used in clinical practice. Still, they may have better responses to palbociclib and letrozole in the first line of the metastatic setting [30]. This result is consistent with recently published series [30,31] and preliminary reports [32,33,34].

These results suggest that a significant proportion of patients with metastatic HR+ HER2− breast cancer in Chile may benefit from targeted therapies for PIK3CA and HER2. The high cost of molecular diagnostics and newer cancer medications remain a significant challenge in Latin America. The financial burden on healthcare systems is substantial, and the disparity in access is exacerbated by economic, political, and ethical issues [35]. A review by Ruiz et al. [36] highlighted the rising cost of drugs, with targeted therapy accounting for a growing proportion of oncology drug expenditure. Additionally, Moye-Holz and Vogler [37] found that cancer treatment is largely unaffordable in Latin American countries, with prices exceeding the financial capabilities of patients and providers. During the last decade, access to genomic medicine has become a part of health equity [38]. High-income countries, such as the United Kingdom, France, Australia, Saudi Arabia, and Singapore have developed national networks aiming to solve the need for advanced molecular testing, generation of representative genomic/genetic data, and harmonization of methods and protocols [39,40]. There, governments worked together with healthcare providers and scientists to establish a sustainable framework to operate in the long term. While a short-term solution will not be available in Latin America, governmental entities may start early efforts by assessing human capital, local technical resources, patient data management, demographics, and geographical variables required to implement such systems.

Our study is not without limitations. The most important is the retrospective nature of our series. While nucleic acids can be confidently extracted from archival FFPE tissues [41] if adequate preanalytical conditions are met, a lack of uniformity and missing data were observed while searching records in three hospitals. Also, this type of research is prone to selection and historical bias due to the availability of samples and changes in the standard of care over time. Finally, we limited this study to PIK3CA and did not expand on other genomic biomarkers. Among the strengths of our research, we can count the size of our series, dedicated to a single subtype of metastatic breast cancer. Also, a diverse population was tested, coming from public and private institutions, showing real-world data. Finally, all cases were centrally tested.

In conclusion, we provide valuable information into the molecular landscape of advanced HR+ HER2− breast cancer in Chile, offering one of the first in-depth analyses of PIK3CA mutations in our population. The insights from our study have the potential to inform clinical decision-making and improve personalized treatment strategies in Chile, addressing a significant gap in the availability of local data on targeted therapies for breast cancer. As precision oncology continues to evolve, our findings underscore the importance of integrating molecular diagnostics into routine practice to enhance patient outcomes.

## 4. Materials and Methods

### 4.1. Patients

We retrospectively collected 102 formalin-fixed, paraffin-embedded (FFPE) metastatic samples from 96 patients at three tertiary hospitals in Santiago, Chile (Clinica Alemana de Santiago, Hospital Dr. Luis Tisné, and Hospital Dr. Sotero del Río). All patients were diagnosed with metastatic breast carcinoma (stage IV, metastatic, AJCC 8th edition) between 2007 and 2023, and their primary tumors expressed ≥1% nuclear positivity for estrogen and/or progesterone receptors [42]. Patients whose metastasis tested positive for hormone receptors by immunohistochemistry were included if information on the primary tumor was unavailable. All patients were over 18 years old at the time of diagnosis. A retrospective review of the patient’s pathology reports was conducted, and the following characteristics were recorded: age at stage IV diagnosis, metastatic site, and immunohistochemical pattern for estrogen receptor, progesterone receptor, and HER2. A search was conducted in the Chilean Civil Registry to determine the date of death of the corresponding patients. This study was approved by the institutional Ethics Committee of Clinica Alemana School of Medicine at Universidad del Desarrollo and locally at other centers.

### 4.2. Tissue Selection and Nucleic Acid Extraction

Formalin-fixed, paraffin-embedded tissue samples were stained with hematoxylin and eosin, reviewed, and marked by a breast pathologist. Only samples with a minimum of 10% tumor content in the marked area were selected for this study. Tumors with high necrosis and fewer than 500 tumor cells in the chosen area were excluded. Stained tissues were macrodissected to select tumor cell-enriched areas, from which genetic material was extracted using a RecoverAll Total Nucleic Acid Isolation Kit for FFPE (Thermo Fisher, Waltham, MA, USA, AM1975) according to the manufacturer’s protocol. The nucleic acids (DNA) were quantified using a Qubit 2.0 fluorometer (Qubit dsDNA HS Assay Kit, Thermo Fisher, Q32851) following the manufacturer’s specifications.

### 4.3. Sequencing Panel, Library Preparation, and Template Preparation

Samples were analyzed using various gene panels, including mutational hotspots or the entire coding sequence of PIK3CA. For assays, 20 ng of genomic DNA was used. Ion AmpliSeq Library Kit 2.0 (Thermo Fisher, 4475345) and Ion Xpress Barcode Adapters kit (Thermo Fisher, 4471250, 4474009) were employed for library preparation according to the manufacturer’s instructions. Library quantification was performed with Ion Library Quantitation kit (Thermo Fisher, 4468802). Multiplexing was conducted, each at a 100 pM concentration, in preparation for the PCR emulsion step utilizing Ion OneTouch 2 System and Ion PGM Hi-Q OT2 Kit (Thermo Fisher, A29900).

For the sequencing process, Ion PGM Hi-Q Sequencing Kit (Thermo Fisher, A25592) was utilized, and the samples were loaded onto an Ion 318 v2 chip (Thermo Fisher, 4488146) using Ion Personal Genome Machine (PGM, Thermo Fisher).

### 4.4. Data Analysis

The Torrent Suite platform and Ion Reporter software for DNA (version 5.20, Thermo Fisher, Waltham, MA, USA) were used for sequencing analysis. A coverage of >250 reads was required for variant calling, with a 5% allelic frequency cutoff for known SNVs/mutations and 10% for known indels as reported in COSMIC. The human genome hg19 was used as a reference for read alignment. Only pathogenic mutations were obtained.

### 4.5. Statistical Analysis

For analysis of clinicopathological characteristics, a Fisher´s exact test was used. For survival, we used a log-rank test and Kaplan–Meier estimator. All tests were performed using GraphPad Prism 10 (GraphPad Software, Boston, MA, USA) and considered statistically significant if *p* < 0.05.

### 4.6. Lollipop Plot

Lollipop plots allow the visualization of variants in a linear chart of the protein. We accessed the MutationMapper tool (University of Oxford, Oxford, UK) in cBioPortal [43,44]. Mutation data were input using protein nomenclature. A graphic was generated including the distribution and quantity of mutations in the PIK3CA protein.

### 4.7. Access to Publicly Available Genomic Data

As of 2024, public databases with genomic information from metastatic tissues from hormone receptor-positive breast cancer patients are not available. The Metastatic Breast Cancer (MBC) Project [27] is a repository containing exomic and transcriptomic data from tumor tissues from primarily early-stage patients, some of them with known metastatic progression. Information is currently indexed in cBioPortal. We extracted selected clinico-pathological data from 70 hormone receptor-positive patients, including known metastatic sites (ever in their evolution) and pathogenic PIK3CA mutations.

## Figures and Tables

**Table 1:** Clinicopathological characteristics of the Chilean metastatic hormone receptor-positive breast cancer series.

		N = 96 Patients	N = 102 Samples	%
Age at stage IV dg (years)	<50	25		26
	≥50	65		67.7
	Unknown	6		6.3
Stage IV at diagnosis	Yes	23		24
	No	68		70.8
	Unknown	5		5.2
Metastatic site	Liver		30	29.4
	Bone		18	17.6
	Lung/Pleura		17	16.7
	Soft tissue		13	12.7
	Skin		9	8.8
	Other		15	14.7
Metastatic IHC pattern	HR+, HER2−	80	85	83.3/83.3
	HR+, HER2+	6	6	6.3/5.9
	HR−, HER2−	6	7	6.3/6.9
	HR−, HER2+	4	4	4.2/3.9
Breakdown HER2-IHC	HER2-low	55	61	57.3/59.8
	HER2-ultralow	18	18	18.8/17.6
	HER2 status only	13	13	13.5/12.7
PIK3CA mutations	Exon 1	2	2	2.1/2
	Exon 4	3	3	3.1/2.9
	Exon 7	2	2	2.1/2
	Exon 9	14	15	14.9/14.7
	Exon 20	16	16	16.7/15.7
	Exon 7 + 9	1	1	1/1
	Exon 7 + 20	1	1	1/1
	Exon 9 + 13	1 *	1	1/1
	Total mutated	39	41	40.6/40.2
Metastatic follow-up (months)	Median	28.7		
	Range	0.3–181.3		

* The patient with PIK3CA exon 9 + 13 mutations had exon 9 mutations in previous samples and was counted once.

**Table 2:** Clinicopathological characteristics according to PIK3CA mutational status in metastatic hormone receptor-positive breast cancer patients.

		PIK3CA-MUT	PIK3CA-WT	*p*-Value
Age at stage IV dg (years)	<50	10	15	0.8
	≥50	28	37	
Stage IV at diagnosis	Yes	9	14	0.8
	No	29	39	
Metastatic site	Liver	14	13	0.2
	Bone	8	10	
	Lung/Pleura	3	12	
	Soft tissue	5	9	
HER2 status	HER2-low	20	35	0.2
	HER2-ultralow	10	8	

**Table 3:** Comparison between Chilean MHRPBC series and patients from Metastatic Breast Cancer Project.

	Total Patients	Metastatic Sites (%)	PIK3CA Mutated (%)	Exons Involved (%)
Araya et al.	96	Liver (29.4)	39 (40.6)	Exon 1 (5.1)
		Bone (17.6)		Exon 4 (7.7)
		Lung/pleura (16.7)		Exon 7 (5.1)
				Exon 9 (38.5)
				Exon 20 (41)
MBC Project	70	Liver (35.7)	19 (27.1)	Exon 1 (5.3)
		Bone (68.6)		Exon 9 (57.9)
		Lung/pleura (31.4)		Exon 20 (36.8)

## Data Availability

Deidentified patient and molecular data are locally available due to an Internal Review Board decision. Information may be shared after formal petition by investigators.
